# Effects of two alfalfa preparations with different particle sizes on the gastric mucosa in weanlings: alfalfa chaff versus alfalfa pellets

**DOI:** 10.1186/s12917-016-0733-5

**Published:** 2016-06-14

**Authors:** Sarah Vondran, Monica Venner, Ingrid Vervuert

**Affiliations:** Institute of Animal Nutrition, Nutrition Diseases and Dietetics, Faculty of Veterinary Medicine, University of Leipzig, D-04103 Leipzig, Germany; Equine Veterinary Clinic, D-38162 Destedt, Germany

**Keywords:** Squamous, Glandular, Stomach, Gastric lesions, Horse

## Abstract

**Background:**

Feeding alfalfa hay is often recommended for its buffering components, like protein and calcium, to prevent lesions of the gastric mucosa in horses. Until now, there has been no information regarding the influence of alfalfa particle size on the gastric mucosa. The aim of this study was to investigate the effects of feeding two alfalfa preparations with different particle sizes (alfalfa chaff vs alfalfa pellets) in comparison with grass hay on the gastric mucosa in weanling horses. We hypothesized that feeding a high proportion of fine alfalfa particles would negatively impact gastric mucosa and that feeding long alfalfa chaff would improve gastric mucosal health in weanlings.

**Results:**

Before weaning, the prevalence of gastric mucosa lesions (one or more lesions considering all locations in the stomach) was 84.3 %; at 14 days after weaning, it was almost 100 %. Before and after weaning, most of the lesions were found at the greater curvature of the squamous mucosa and at the lesser curvature. After weaning, gastric mucosal lesions at the pylorus were significantly more severe in the group fed alfalfa chaff (*p* = 0.002). In the other regions, no differences related to the feeding regimes were observed.

**Conclusions:**

Feeding alfalfa failed to improve gastric mucosal lesion scores in weanlings. Furthermore, foals fed alfalfa chaff had higher lesion scores at the pylorus. Alfalfa leaves contain a superior protein source and high amounts of calcium and magnesium, providing extra nutritional advantages in growing horses. At this time, either traditional grass hay rations or grass hay with alfalfa pellets can be recommended.

## Background

Gastric ulceration is commonly identified in horses and foals. The prevalence of gastric ulcers in Thoroughbred racehorses is estimated to be >80 % in the squamous mucosa [1]. In weanlings it ranges between 32 and 94 % [2, 3]. Risk factors for gastric mucosa lesions, specifically for equine squamous gastric ulcer disease, are stall confinement, strenuous exercise, transport stress in adult horses (as reviewed by Andrews and others [4]), and the weaning process in foals [2, 3]. Nonsteroidal anti-inflammatory drugs have been demonstrated to be a risk factor for the equine glandular region in adult horses and foals [5, 6].

The relationship between feeding and gastric mucosal health has been demonstrated. Dietary factors that may effect squamous gastric mucosa in horses include buffering substances [7], the level of starch intake [8], or the type and daily amount of food [8, 9]. Furthermore, the particle size of the diet appears to be a factor in glandular gastric mucosa [2]. In foals, feeding alfalfa chaff resulted in glandular mucosal lesions, which may have been related to mechanical irritation [2]. Furthermore, no favorable effect was found on the squamous mucosa in weanlings. However, other authors observed beneficial effects of feeding alfalfa hay on the squamous mucosa in foals [10] and adult horses [7]. In pigs fed a diet of finely ground pellets, the squamous gastric ulcer score was significantly higher than for those fed a diet of larger particles [11]. Furthermore, feeding a coarse ground feed reduced mucosa lesions of the pars oesophagea in growing pigs [12].

The aim of this study was to investigate the effects of feeding two alfalfa preparations with different particle sizes (alfalfa chaff vs alfalfa pellets) in comparison with grass hay on the gastric mucosa in weanling horses. We hypothesized that feeding a high proportion of fine particles (alfalfa pellets) would negatively impact gastric mucosa in foals and that foals receiving alfalfa chaff would have better gastric mucosal lesion scores.

## Results

None of the foals in the study demonstrated any clinical signs commonly associated with the presence of gastric ulcers such as colic, inappetence, or depression, as reviewed by Andrews and others [4].

The daily average consumption of grass hay per foal was 4.3 kg in the alfalfa chaff group, 5.0 kg in the alfalfa pellets group, and 8.3 kg in the grass hay group (Table 1). In addition to the grass hay intake, the mean daily feed intake (± SD) was 5.95 ± 0.13 kg in the alfalfa chaff group, 5.47 ± 0.18 kg in the alfalfa pellets group, and 3.66 kg in the hay group.

**Table 1:** Mean (± SD) daily dry matter and nutrient intake for each foal according to diet

Diet	DM	Ash	CP	CF	NDF	Starch	Ca	P	Mg
AC	9383 ± 120	461 ± 9	1190 ± 19.5	2978 ± 54	5265 ± 75	1410 ± 7	82 ± 1.6	27 ± 0.4	16 ± 0.2
AP	9798 ± 167	608 ± 21	1232 ± 31	2937 ± 54	5585 ± 79	1410 ± 9.5	86 ± 4	26 ± 0.4	16 ± 0.3
HA	10,800	457	1270	3286	6729	1404	89	29	20

Intake is expressed as grams

AC: diet including 3 kg alfalfa chaff, 4.3 kg grass hay, 2.7 kg oats, 240 g soybean meal, 70 g calcium oxide and 40 g of a commercial trace element mixture; AP: diet including 3 kg alfalfa pellets, 5.0 kg grass hay, 2.7 kg oats, and 40 g of a commercial trace element mixture; HA: diet including 8.3 kg grass hay, 3 kg oats, 0.5 kg soybean meal, 120 g CaCO_3_ and 40 g of a commercial trace element mixture

*Ca* calcium, *DM* dry matter, CF crude fiber, *CP* crude protein, *HA* diet including hay, *Mg* magnesium, *NDF* neutral detergent fiber, *P* phosphorus

In the alfalfa chaff group, 91 % of alfalfa chaff particles were longer than 2 mm; in the alfalfa pellets group, 60 % of alfalfa pellets particles were smaller than 0.25 mm (Table 2).

**Table 2:** Percentages of different particle sizes of alfalfa pellets and alfalfa chaff

Feedstuff	>2 mm	>1 mm	>0.5 mm	>0.25 mm	<0.25 mm
Alfalfa chaff	90.8 %	6.62 %	1.94 %	0.28 %	0.31 %
Alfalfa pellets	4.63 %	6.95 %	12.4 %	16.1 %	59.9 %

Mean (± SD) body weight increased in all groups (alfalfa chaff group: 4.8 ± 1.9 %; alfalfa pellets group: 5.5 ± 2.5 %; hay group: 4.3 ± 2.3 %). There was no significant difference (*p* = 0.49) in weight gain between groups.

**Fig. 1:**
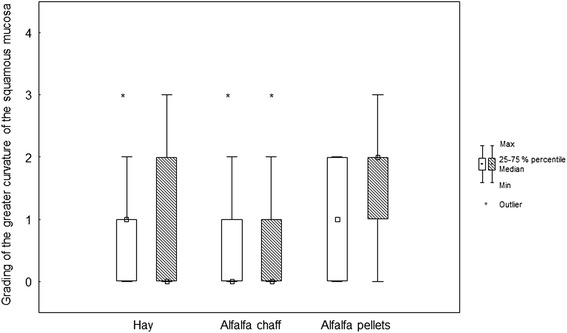
Box (median) and whisker (25th/75th percentiles) plots of lesions grades for the greater curvature of the squamous mucosa before and after weaning for the different feeding protocols (white plots before weaning; striped plots after weaning)

**Fig. 2:**
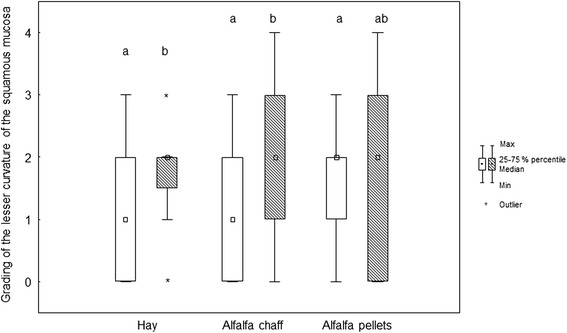
Box (median) and whisker (25th/75th percentiles) plots of lesions grades for the lesser curvature of the squamous mucosa before and after weaning for the different feeding protocols (white plots before weaning; striped plots after weaning). Unlike letters are different with *p* < 0.05

**Fig. 3:**
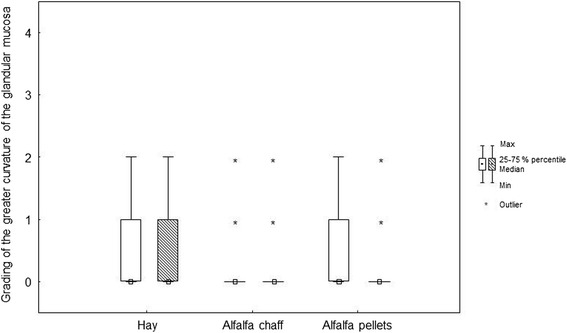
Box (median) and whisker (25th/75th percentiles) plots of lesions grades for the greater curvature of the glandular mucosa before and after weaning for the different feeding protocols (white plots before weaning; striped plots after weaning)

**Fig. 4:**
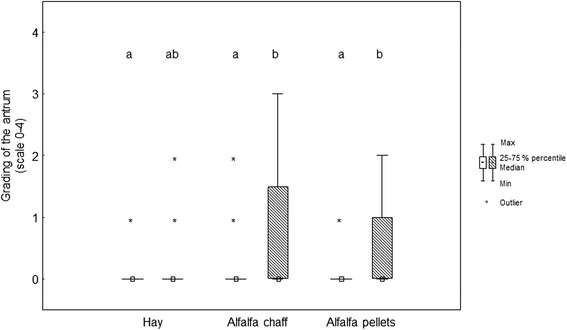
Box (median) and whisker (25th/75th percentiles) plots of lesions grades for the antrum before and after weaning for the different feeding protocols (white plots before weaning; striped plots after weaning). Unlike letters are different with *p* < 0.05

**Fig. 5:**
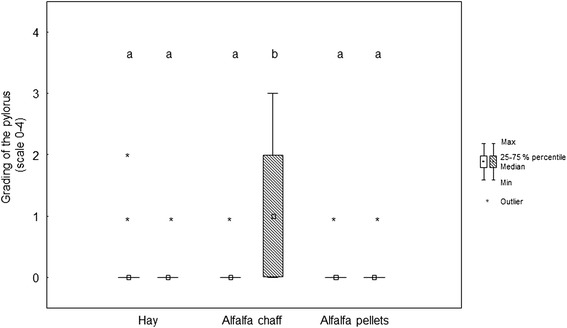
Box (median) and whisker (25th/75th percentiles) plots of lesions grades for the pylorus before and after weaning for the different feeding protocols (white plots before weaning; striped plots after weaning). Unlike letters are different with *p* < 0.05

Before weaning (*T* = 0), the prevalence of gastric mucosa lesions (one or more lesions considering all locations in the stomach) was 84.3 %; at 14 days after weaning (*T* = 16), it was almost 100 %. Before and after weaning, most of the lesions were found at the greater curvature of the squamous mucosa and at the lesser curvature (Table 3, Figs. 1, 2, 3, 4 and 5).

**Table 3:** Grading of the different stomach regions before weaning for the different feeding protocols

Diet	Squamous region			Glandular region		
	Dorsal fundus	Lesser curvature	Greater curvature	Greater curvature	Antrum	Pylorus
AC	0 (0/0)	1 (0/2)	0 (0/1)	0 (0/0)	0 (0/0)	0 (0/0)
AP	0 (0/0)	2 (1/2)	1 (0/2)	0 (0/1)	0 (0/0)	0 (0/0)
HA	0 (0/0)	1 (0/2)	1 (0/1)	0 (0/1)	0 (0/0)	0 (0/0)
p value	0.39	0.20	0.18	0.31	0.25	0.94

Data expressed as median (25th/75th percentiles)

### Effect of weaning on gastric mucosa

Only very few foals had gastric mucosa lesions in the dorsal squamous fundus before and after weaning in the three diet groups. In the squamous region of the greater curvature, the median lesion score was 1 (0/1) before and 0 (0/2) after weaning in the hay group (*p* = 0.78), 0 (0/1) before and 0 (0/1) after weaning in the alfalfa chaff group (*p* = 0.21), and 1 (0/2) before and 2 (1/2) after weaning in the alfalfa pellets group (*p* = 0.17) (Fig. 1). In the squamous region of the lesser curvature, the median lesion score increased from 1 (0/2) before to 2 (1.75/2) after weaning in the hay group (*p* = 0.03), increased from 1 (0/2) before to 2 (1/3) after weaning in the alfalfa chaff group (*p* = 0.02), and was 2 (1/2) before and 2 (0/3) after weaning in the alfalfa pellets group (*p* = 0.14) (Fig. 2).

In the glandular region of the greater curvature, the median lesion score was 0 (0/1) before and 0 (0/1) after weaning in the hay group (*p* = 1), 0 (0/0) before and 0 (0/0) after weaning in the alfalfa chaff group (*p* = 0.48), and 0 (0/1) before and 0 (0/0) after weaning in the alfalfa pellets group (*p* = 0.35) (Fig. 3). In the antrum, the median lesion score was 0 (0/0) before and 0 (0/0) after weaning in the hay group (*p* = 0.18), 0 (0/0) before and 0 (0/1) after weaning in the alfalfa chaff group (*p* = 0.01), and 0 (0/0) before and 0 (0/1) after weaning in the alfalfa pellets group (*p* = 0.03) (Fig. 4). At the pylorus, the median lesion score was 0 (0/0) before and 0 (0/0) after weaning in the hay group (*p* = 1), 0 (0/0) before and 1 (0/2) after weaning in the alfalfa chaff group (*p* < 0.001), and 0 (0/0) before and 0 (0/0) after weaning in the alfalfa pellets group (*p* = 0.56) (Fig. 5).

### Effect of diet on gastric mucosa

Before weaning (*T* = 0), the gastroscopic findings of each region did not differ significantly between the three diet groups (Table 3). After weaning (*T* = 16), gastric mucosal lesions at the pylorus were significantly more severe in the group fed alfalfa chaff (*p* = 0.002) (Fig. 5). In the other regions, no differences related to the feeding regimes were observed.

## Discussion

Various methods have been used to induce gastric ulcers. In this study we used the weaning process as a model to induce gastric ulcers in foals to determine the effects of feeding. Previous studies have shown that the weaning process is a stressful event in a foal’s life and may increase gastric lesions. Dahlkamp and others [3] reported that ulcer scores of the squamous region, as examined by gastroscopy, increased from 48 % before weaning to 78 % 2 weeks after weaning. This study revealed an initial prevalence of gastric lesions in glandular and squamous regions of 84.3 % prior to weaning. The changes in feeding and housing management 1 week before weaning may have resulted in high gastric lesion scores. The introduction to new stables and housing groups was performed to ensure standardized experimental conditions.

The aim of the study was to evaluate the impact of feeding different particle sizes to weanlings. Gastric findings of each region of the stomach were scored separately because we assumed that diet regime might have various influences on the gastric mucosa of different regions.

One food source recommended to prevent gastric lesions in horses is alfalfa hay. Nadeau and others [7] found a lower incidence of gastric lesions in horses fed alfalfa hay. The reason for the beneficial effects of feeding alfalfa is related to its buffering components, like protein and calcium. High calcium intake reduced the basal HCl secretion in rats. This effect was suspected to be a result of higher extracellular and intracellular calcium concentrations in parietal and G cells. Higher extracellular and intracellular calcium concentrations may lower the cAMP concentration, thus reducing the production of HCl [13]. Exposure of the equine gastric mucosa to HCl and short-chain fatty acids significantly decreased sodium transport of the cells. The addition of calcium carbonate decreased the sodium transport to baseline [14]. From these results, the authors proposed that calcium has a protective effect in the development of gastric lesions. Nadeau and others [7] found significantly lower scores of the squamous mucosa in horses fed an alfalfa hay/grain diet compared to horses fed a brome hay diet. The authors concluded that high amounts of protein and the quality of the protein may have buffering effects in the stomach, as previously seen in cattle [15].

Alfalfa is believed to have beneficial effects on gastric mucosa by buffering gastric pH because it contains large amounts of calcium, magnesium, and protein. In the present study, calcium and protein intake were approximately equal among the different feeding groups (Table 1), but particle size between the two alfalfa preparations was different. Former studies have demonstrated the impact of the particle size on the stomach mucosa in different species. Saliva production is related to the particle structure of a diet, and saliva has a buffering effect due to its potassium, chloride, and bicarbonate [16]. For example, feeding 1 kg of hay results in 3 to 6 L of saliva production and feeding 1 kg of grain results in 1 to 1.7 L of saliva production in adult horses [17]. The concentration of bicarbonate in saliva may impact stomach pH [18]. These effects may explain the lower gastric lesion scores in weanlings fed hay in our study. However, lesion scores of weanlings in the alfalfa pellets group were not significantly different from those in the hay group. It is possible that although the foals fed alfalfa pellets may have produced less saliva, the high-quality protein in the alfalfa may have resulted in ulcer scores similar to those in the hay group.

Foals fed alfalfa chaff had higher ulcer scores at the pylorus than those fed alfalfa pellets or hay. These results were surprising because alfalfa chaff has been suggested to be associated with higher saliva production due to the more intensive chewing involved; increased saliva flow is known to have a buffering effect on the stomach. The findings in the alfalfa chaff group were similar to those of Fedtke and others [2]. These authors noted that feeding alfalfa chaff resulted in significantly greater ulcer scores at the pylorus following weaning. They speculated that the pyloric ulcers might be related to the small particles of the alfalfa chaff, similar to results obtained in swine [2]. Mößeler and others [11] fed pigs four different diets with different grinding intensities and different physical forms (pellets vs meal) for 3 days. Animals were examined post-mortem. Samples of the frozen gastric contents were taken from 15 standardized locations of the stomach. Mößeler and others [11] demonstrated that a finely ground pellet diet resulted in more liquid gastric contents and that pH values were similar in all regions of the stomach (mean ± SD pH difference between the regions: 0.175 ± 0.140). In contrast, a coarsely ground diet resulted in stratification of the gastric chyme content, with a pH difference of 2.22 ± 1.04 (mean ± SD). In the squamous region, the gastric ulcers score was significantly higher in pigs fed a finely ground pellet diet in comparison with pigs fed a coarsely ground diet without pellets [19]. All studies performed on pigs focused on the squamous region, which is the most common region for development of gastric ulceration in pigs [20]. Pigs fed a diet of finely ground pellets also showed greater histological evidence of inflammation compared to pigs fed other diets [21].

However, our results differed from those noted in pigs. Higher ulcer scores were observed after feeding alfalfa chaff compared to hay or alfalfa pellets. In our study alfalfa pellets with a particle size smaller than 1 mm did not induce pyloric lesions. This might be due to the fact that all foals in our study had free access to hay and straw bedding as sources of food. This factor may have masked the negative effects of fine particles in the diet. However, due to the large sample size number, we could not provide individual feeding, but due to the very close monitoring of the foals, we can ensure a similar feed intake within the groups and between the different diets.

It is possible that the harsh acanthous structure of alfalfa chaff may cause mechanical injury at the pylorus. The pyloric region is an area of the stomach with high motility. We assume that the movement of the alfalfa stems may excoriate the mucosa during passage. This initial injury of the glandular mucosa might leave the pylorus more vulnerable to the insults of short-chain fatty acids, hydrochloric acid, and/or bile salts. Pyloric lesions are of particular clinical concern because damage to the pylorus may result in pyloric stenosis with delayed gastric emptying [22]. However, further studies are required to determine whether feeding the whole alfalfa plant may result in similar findings.

## Conclusions

Foals fed alfalfa chaff had higher lesion scores at the pylorus. These findings stress the importance of scoring all gastric regions separately. Furthermore, in the past, particle size has been neglected in equine nutrition; however, our data seem to show this might be an issue in the occurrence of pyloric lesions. It remains unclear whether the mucosa injuries can be observed only in weanlings during the stressful weaning process as a result of impaired mucosal defence or whether the damage can also be induced in adult horses. Nevertheless, alfalfa leaves contain a superior protein source and high amounts of calcium and magnesium [23], providing extra nutritional advantages in growing horses. At this time, either traditional grass hay rations or grass hay with alfalfa pellets can be recommended.

## Methods

### Animals

Seventy warmblood foals (39 males, 31 females), 164 to 234 days of age, with a mean body weight (± SD) of 256 ± 24 kg were included in the study. All foals were born and raised at a stud farm in Germany. Inclusion criteria for the foals were minimum age of 150 days, no findings at clinical examination and physiological white blood cell count, and no medication 10 days prior to initiation of the study. At T = -7, the foals and mares were moved into covered barns with straw bedding and free access to concrete runs. Mares and foals were fed a total mixed ration (TMR) once per day (approximately 13 kg per mare and foal per day) to meet or exceed the energy and nutrient requirements of the mares. The TMR comprised 3 kg corn silage, 6 kg grass silage, 2 kg oats, 0.5 kg straw, 0.3 kg soybean meal, 0.05 kg of a commercial mineral vitamin supplement (Vilomix Biolex; Deutsche Vilomix Tierernährung GmbH, Neuenkirchen-Vörden, Germany), and 0.04 kg CaCO_3_. Horses had access to grass hay and water ad libitum.

Foals were weaned at *T* = 0 and grouped with respect to their dietary treatment. All groups were housed as described. Sample size calculation was performed with PASS 2002 (NCSS, Statistical Software). The required sample size was 15 foals. To ensure a proper sample size at the end of study, and taking into account that some foals might have to be excluded during the study period due to reasons not related to the study design, we increased the groups to up to 25 foals. During the study we had to exclude five foals because of orthopedic injuries. Foals were allocated to each group according to the homogenous distribution of gastric findings at the first gastroscopy.

During the study period the foals were monitored three times daily by a veterinarian for signs of discomfort, depression, inappetence, bruxism, or colic.

### Feeding

Before weaning, foals were introduced to their respective diets from *T* = -3 to *T* = 0 (day of weaning) without being separated from their dam.

After weaning, the foals were separated from their dam and allocated to the three diets. In the alfalfa chaff group (*n* = 25), foals were fed daily with 3 kg alfalfa chaff per foal and had free access to grass hay. Additionally, foals were offered 2.7 kg oats, 240 g soybean meal, 70 g calcium oxide and 40 g of a commercial trace element mixture (see above) mixed with the alfalfa chaff. To ensure uniform consumption, alfalfa chaff was mixed with 400 ml of water for each foal to bind alfalfa chaff to the other feed components.

In the alfalfa pellets group (*n* = 21), each foal was fed daily with 3 kg alfalfa pellets composed of finely ground alfalfa leaves. Additionally, each foal was fed daily with 2.7 kg oats and 40 g of a commercial trace element mixture (see above) and had free access to grass hay. The alfalfa pellets were mixed with oats and the commercial trace element mixture.

The hay group (*n* = 24) had free access to grass hay and received 3 kg oats, 0.5 kg soybean meal, 120 g CaCO_3_ and 40 g of a commercial trace element mixture (see above).

Each diet was standardized to supply a similar nutrient intake per day in each feeding group. The feed was offered in two equal portions twice daily (7:00 AM and 3:30 PM) in a continuous trough providing enough room for each foal. Feed intake was monitored several times daily and remaining feed was weighed after 24 h. In total, the different diets were fed for 16 days after the weaning process. The nutrient intake is summarized in Table 1.

### Gastroscopy

A gastroscopy with a flexible gastroscope (Karl Storz, Tuttlingen, Germany) with a working length of 3 m was performed immediately prior to weaning (*T* = 0) and again after the feeding period of 16 days (*T* = 16). Prior to gastroscopy, foals were fasted by using muzzles for 9 to 12 h, but they had water ad libitum. The foals were sedated with 0.02 mg/kg body weight intravenous detomidinhydrochloride (Cepesedan®; CP Pharma, Burgdorf, Germany). The stomach was insufflated with air until the mucosal folds vanished. The following regions of the stomach were evaluated separately: dorsal squamous fundus; lesser curvature; greater curvature of the squamous region; greater curvature of the glandular region; antrum; and pylorus. Each region and mucosal type was given a score from 0 to 4. Squamous mucosa was scored according to a proposal of the European College of Equine Internal Medicine [24] (Table 4). Glandular gastric mucosa including the antrum and pylorus was evaluated according to a modified scoring system (Table 5). Gastric mucosa was evaluated during gastroscopy by the same blinded and experienced investigator (MV).

**Table 4:** Scoring system of the squamous regions of the equine stomach. Adapted from the proposal of the European College of Equine Internal Medicine [24]

Grade	Characteristics
0	Epithelium intact and no appearance of hyperkeratosis
1	Mucosa intact, but areas of hyperkeratosis
2	Small, single or multifocal lesions
3	Large single or extensive superficial lesions
4	Extensive lesions with areas of apparent deep ulceration

**Table 5:** Scoring system of the glandular regions including the antrum and pylorus of the equine stomach

Grade	Characteristics
0	Epithelium intact and no appearance of hyperemia (reddening) or fibrinosupperative areas
1	Intact flat mucosa, but with small single or multifocal areas of reddening
2	Raised mucosa with large single or multifocal areas of reddening or fibrinosupperative areas, no signs of bleeding
3	Raised mucosa with hemorrhagic and fibrinosupperative areas
4	Ridged or depressed mucosa with severe signs of bleeding or with large and distinct fibrinosupperative areas

### Scaling

Body weight was measured by an electronic weight scale (Müller, Jagstzell, Germany) immediately before each gastroscopy. Feedstuffs were weighed using an automatic scale (Beurer, Ulm, Germany and Waagen-Schmitt GmbH, Hamburg, Germany).

### Nutrient analysis

Dry matter (DM) was determined after oven-drying (103 °C) to a constant mass (Table 6). Crude ash was measured by ashing the feeds in a muffle furnace (6 h, 600 °C) [25]. Starch content was estimated polarimetrically (Polartronic E; Schmidt and Haensch, Berlin, Germany). Sugar contents (glucose and sucrose) were determined by the Luff Schoorl method. Crude nutrients were assayed by the Weende system [25]. Neutral detergent fiber was analyzed by the Fibertec® (Tectator, Rellingen, Germany) [26].

**Table 6:** Chemical composition of feed

		Alfalfa chaff	Alfalfa pellets	Hay	Oats
Ash	g/kg DM	71	117	45	26
CP	g/kg DM	150	172	95	123
CF	g/kg DM	412	302	372	132
NDF	g/kg DM	578	441	732	355
Ca	g/kg DM	12.3	20.9	5.2	1.2
P	g/kg DM	2.74	2.3	1.8	4.6
Mg	g/kg DM	1.65	1.8	1.7	1.7

*Ca* calcium, CF crude fiber, *CP* crude protein, *DM* dry matter, *NFE* nitrogen-free extractives, *Mg* magnesium, *P* phosphorus

### Sieving

To determine particle sizes, 50 g of alfalfa pellets were soaked with 1 L water for 24 h prior to sieving. Samples were sieved for 5 min with an extrusion of 1.5 mm and running water. Samples were dried (60 °C) before weighing. Fifty grams of dry alfalfa chaff were sieved for 5 min with an extrusion of 1 mm. Sizes of the sieves were 2 mm, 1 mm, 0.5 mm, and 0.25 mm.

### Statistical analysis

Statistical analysis was performed using a statistical software program (STATISTIKA, StatSoft). Body weight was analyzed for normal distribution by the Shapiro-Wilk W test. Stomach scores were calculated using medians, 25^th^ and 75^th^ percentiles, and ranges. The Wilcoxon signed rank test was used to compare gastroscopic scores within a group before and after weaning. Kruskal-Wallis ANOVA was performed to compare the gastroscopic scores between the treatment groups. A value of *p* < 0.05 was considered significant.

## Abbreviations

DM, dry matter; Fig, figure; HCl, hydrochloric cid; L, liter; SD, standard deviation; T, time point; TMR, total mixed ration
